# Incremental Value of Post Diuretic ^68^Ga-PSMA-11 PET-CT in Characterization of Indeterminate lLesions in Prostate Cancer 

**DOI:** 10.31557/APJCP.2020.21.12.3719

**Published:** 2020-12

**Authors:** Layla Ghadanfer, Sharjeel Usmani, Fahad Marafi, Fareeda Al Kandari, Rashid Rasheed

**Affiliations:** 1 *Department of Radiologic Sciences, Kuwait University, Kuwait. *; 2 *Department of Nuclear Medicine, Kuwait Cancer Control Center (KCCC), Kuwait. *; 3 *Sheikh Jaber Al Ahmad Al Sabah for Nuclear Medicine and Molecular Imaging Center, Kuwait.*; 4 *Department of Chemistry, Government College University, Kotwali Rd, Gurunanakpura, Faisalabad, Punjab 38000, Pakistan. *

**Keywords:** Indeterminate lesions, ^68^Ga, PSMA PET/CT, incremental values, post Lasix study, prostate cancer

## Abstract

**Objective:**

The aim of current study is to evaluate the role of diuretic assisted ^68^Ga-PSMA PET-CT, on image quality and clinical interpretation of indeterminate/equivocal lesions in pre-Lasix imaging of Prostate cancer.

**Materials and Methods:**

Forty-five patients underwent baseline ^68^Ga-PSMA-11 scan 45-60 minutes post tracer injection followed by post Lasix study after ±15 minutes. The contrast to noise ratios (CNR), noise and SUVmax were determined for the focal uptakes in both pre and post Lasix images. All continuous variables were expressed as mean ± SD. Images were assessed by two experienced physicians in order to evaluate lesion detectability and delineations that have an impact on clinical interpretation.

**Results:**

Of total 45 patients, 12/45 (27%) showed unremarkable scan along with 33/45 (73%) showing metastases. Sixteen out of 45 (36%) of the metastatic scans showed indeterminate/equivocal lesions. In these cases, post Lasix study showed false negative findings in 7/45 (16%), better delineation of lesions 10/45 (22%), better confidence towards reporting lesions as abnormal in 5/45 (11%) with an overall 11/45 (24%) of the cases who showed increase in the number of the lesions after the Lasix study. The overall CNR was evaluated using Wilcoxon Rank test (p-value = 0.02) which suggested significant improved ratios in the post-Lasix imaging by 49.6%±24.5. There was a substantial agreement (k =0.76) between the physicians when comparing the lesion clarity and delineation in post Lasix images. The average score for physician one and two being 2.4 ±0.71 and 2.53±0.52 respectively.

**Conclusion:**

Post diuretic ^68^Ga-PSMA imaging at ± 15 minutes clears the unwanted activity in the urinary tract which in turn improves the contrast to noise ratios. Thus leading to decline in false positive findings, improved diagnostic certainty of physician and better detection of indeterminate lesions in ^68^Ga-PSMA imaging.

## Introduction

Incidence of prostate cancer (PCa) has shown significant incline over past decade and is the most frequent cancer in the men, worldwide (Siegel et al., 2014) with 14.4% of all cases reported in Kuwait in 2018. However, recent cancer statistics in Kuwait show alarmingly 55.3 % increase in the number of patients with PCa by the year 2014, leading to PCa being the top of the list of top ten cancers in Kuwait. 

Despite of advancement in curative management of PCa, biochemical recurrence is frequently evident especially in men with high risk factors (Maurer et al., 2016). In such medical milieu, searching for the subtle recurrent tumor foci in the body is exigent using conventional transmission scans such as CT, MRI or US, especially, when there are very low PSA levels (Herlemann et al., 2015; Hijazi et al., 2015). In such scenarios, of the current decade, functional imaging using targeted prostate specific membrane antigen (PSMA) inhibitor molecules (18F-PSMA or ^68^Ga-PSMA-11) have shown enormous potential for hunting imperceptible tumor foci (Pyka et al., 2016; Ali Oromieh et al., 2017). ^68^Ga-PSMA-11 have shown better imaging qualities when compared to other PET-CT tracers for molecular imaging in PCa i.e. C-11 or 18F-choline, C-11 acetate and 13N-ammonia (Eiber et al., 2015; Chang et al., 2016).

^68^Ga-PSMA-11 PET-CT imaging show high target to back ground ratios as the hydrophilic tracer is actively excreted through the kidneys. Although, PSMA show low non-specific uptake in the neo-vasculature of non-prostate tissues i.e. breast, liver, brain etc. the physician encounters the most often artifacts in the urinary tract activity during their reporting and hence face difficulty in characterization of the lesions. 

Despite of specificity of PSMA, renal route of excretion pose difficulty in the interpretation and characterization of the lesions in the abdomen and pelvis due to superimposed activity of genitourinary system leading to false positive or false negative reporting, especially in case of possible retroperitoneal and pelvic lymph nodes.

Renal uptake in ^68^Ga-PSMA PET-CT is due to expression of PSMA in the cells of proximal convoluted tubules of the glomerular nephrons (Baccala et al., 2007) leading to intense tracer uptake in kidneys and halo artifacts, posing difficulty in the interpretation of the lesions around its excretion path. In the recent past, different methods were tried to reduce this issue using either delayed 2-3-hour imaging, diuresis with furosemide (Niall Fennessy et al., 2017) or Mannitol infusion (Matteucci et al., 2017).

Aim of current study was to follow the abnormal uptake/lesions presented in pre-Lasix PSMA images and compare them with post-Lasix scan after ±15 minutes in the same patient. The challenge of the presence of abnormalities that might mimic both cancer and urine retention have encouraged us to evaluate the effect of diuretics in ^68^Ga-PSMA imaging and to investigate its impact on the delineation of lesions and thus the accuracy of diagnosis. 

## Materials and Methods

*Study design*

Total of 51 patients were included in the study from December 2017 to December 2018 after the approval of hospital ethical committee. Six out of 51 patients were excluded from our study since they had some contraindications to furosemide i.e. allergic to sulpha medication. Forty-five patients were included in the study with mean age of 61.53 ± 6.46 (range 50 - 73 years) and median age of 63 years. The referrals included initial staging, restaging with recurrent prostate cancer or assessment of response to therapy. The average PSA levels were 43.04 ± 113.41 with a range of 0.003- 440 ng/ml. The inclusion criteria for post Lasix scan included the scans with equivocal lesions, which could not be differentiated from urinary activity on baseline study as per advice of the physician. All other scans without any equivocal activity around the urinary tract were excluded from study. The study was approved by hospital ethical committee and Ministry of Health and an informed consent form was signed by all patients participating the study. All the patients were explained regarding the injection of furosemide and the possibility of repeating low dose CT for the post-Lasix acquisition. 

*Pre-Lasix Imaging*

The pre-Lasix PET-CT was performed Discovery 690 and 710 PET-CT system by GE Healthcare™. The dose administered was according to the body weight i.e. 2.22 Mbq/kg (0.06 mCi/kg). The pre-Lasix study was acquired 60 minutes after the tracer injection followed by supine acquisition from vertex to mid-thigh with hands up position in a 128 x 128 matrix using 2-3 minutes bed per position. Following PET acquisition, a low dose CT scan from the vertex to mid-thigh was acquired for attenuation correction and anatomical localization using smart mAs, 120 KVp, and a matrix size 512x512 with 2.5 mm slice thickness. 

*Post-Lasix Imaging*

For post-Lasix imaging furosemide (Lasix) was given to the patient ± 15 minutes after the baseline scan, according to the patient weight and blood pressure, slowly over 1-2 minutes to prevent any side effect. The blood pressure was continuously monitored, later the patient was instructed to void. Post-Lasix images of the abdomen and pelvis (kidneys, ureters and urinary bladder) were acquiredwith the same parameters that previously used in the pre-Lasix image. 

*Image reconstruction*

PET images were reconstructed using OSEM protocol (subset 8 and 2 iterations) with the time of flight (TOF) and point spread function (PSF) to correct for the variation in resolution across the field of view and also corrected for attenuation using CT attenuation map with 2.5mm slice thickness as per institutional protocol.The number of lesions in Pre and Post Lasix image was recorded and compared in both images. Multiple regions of interest (ROI with the same size) were drawn around the lesions and on the background by simply copy-paste of circular ROIs. Contrast to noise ratio (CNR) was calculated using formula given below;

CNR=(C_l_-C_B_)/σ_B_

Where C_l _is the mean SUV in the lesion, C_B_ is the mean SUV in background ROI and σ_B _is the standard deviation in the background. Noise was also calculated in both pre and post Lasix images using the following formula: 

Noise=σ_B_/A_B_

 Where A_B_ is the count in the background ROI. Maximum standardized uptake (SUVmax) was also compared in both pre and post Lasix image. 

*Image interpretation*

The PET-CT images (45 pre Lasix studies and 45 post Lasix studies) were read by two experience board certified nuclear medicine physicians (with experience of more than 2000 PET/CT reports). Images around urinary system were assessed subjectively on a 3-point scale negative as a lesion (definitive urinary activity), equivocal (suspected lesion or urinary activity), and definitively positive lesion. In case of any discrepancies among the readers a third physician was responsible for resolution of conflict however due to enough experience of physicians no discrepancy was raised what so ever. Although we did low dose CT with slice reconstruction of 2.5 mm as per institutional protocol, however we still used CT to identify an equivocal focus of uptake or potential node close to the ureter and tried to resolve it on the basis of careful review of CT images.

*Statistical analysis*

The scores of the observers were analyzed and the p-value were calculated with the continuity Chi square test and the agreement was tested using Cohen’s Kappa coefficient. A one-way ANOVA test was used to evaluate the semi-quantitative measurements with a P-value < 0.05 considered to be significant. 

## Results

Total 45 patients were included in the study (mean age 61.53 ± 6.46, range 50 - 73 years, median 63) (Table 1). The mean time of imaging after Lasix injection was 15±5min after the baseline scan resulting in mild additional CT dose per patient of 0.99±0.13 mSv. The reconstructed images showed unremarkable noise in pre and post Lasix images. Out of total 45 patients, 12/45 (27%) of the patients were found to have no abnormality in either pre-Lasix baseline scan or post Lasix study. 

Out of rest of the 33/45 (73%) positive cases, 16/45 (36%) of the cases were labeled as indeterminate/equivocal in terms of characterization of lesions in the pre-Lasix study. However, post-diuretic ^68^Ga-PSMA PET-CT data distinguished false positive lesions of 7/45 (16%) in pre-Lasix study, as benign ureteric activity. A better delineation of the lesions was seen in 10/45 (22%) of the post-Lasix images. Out of 16/45 equivocal cases, a better confidence towards reporting of the abnormal lesions was seen in 5/45 (11%) of the cases along with an overall 11/45 (24%) of the cases who showed increase in the number of the lesions after the post-Lasix study. The overall CNR was evaluated using Wilcoxon Rank test which suggested significant improved ratios in the post-Lasix imaging by 49.6 % ±24.5 with a p-value of 0.02.

**Figure 1:**
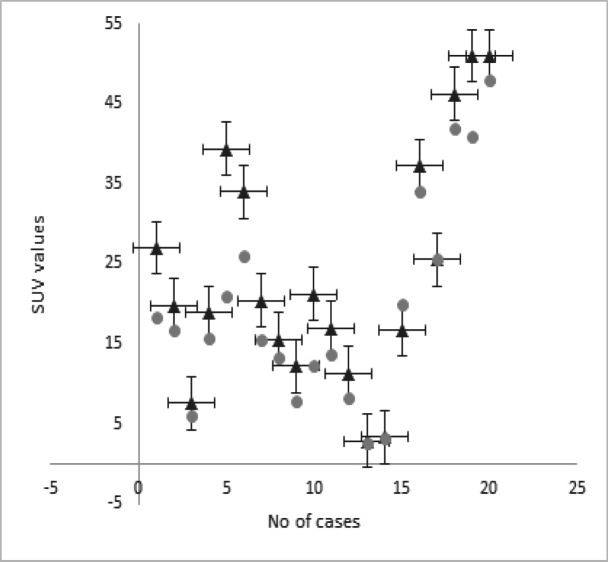
Incline in SUVmax Values Pre-Lasix (Round Circle) and Post-Lasix (Triangles)

There was an acceptable substantial agreement (k =0.76) between the physicians when comparing the lesion clarity and delineation in post-Lasix images, with average score for physician one and two being 2.4 ±0.71 and 2.53±0.52 respectively. SUVmax for lesions observed in post-Lasix image, was significantly increased by an average value of 41% (range, 7.1%-100%) when compared with those found in pre Lasix image (Figure 1). 

The observers pointed out that the interpretation of 36% (n=16) of pre Lasix scans were equivocal which showed accurate diagnostic findings after Lasix study and none of the case out of 45 was left as indeterminate. Both observers agreed that besides better delineation of lymph nodes and prostate lesions, local recurrence and rectal extensions were more obvious and better delineated in the post-Lasix study due to the clearance of ureteric activity. 

**Figure 2:**
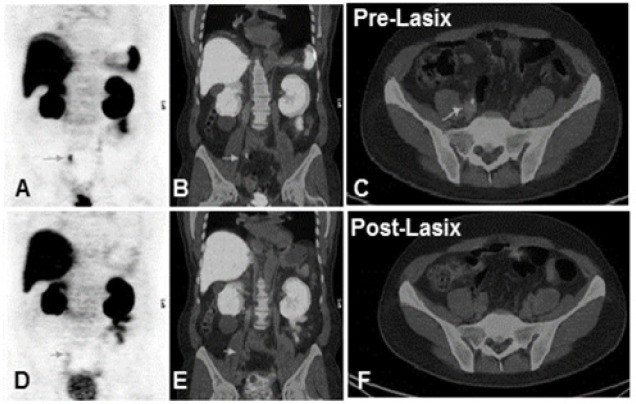
MIP and Fused PET/CT Images (A-C) Respectively, Showing Focal Uptake in the Ureter in Pre Lasix Study (Arrows) which Mimicked PMSA Avid Lesion. However, it was cleared in post Lasix images (D-F)

**Table 1:** Characteristics of Patient Population

Characteristic	Value
Age (years) (n = 45)	
Mean ± SD	61.53±6.46
Median (range)	63 (50 - 73)
Tracer (MBq) (n = 45)	
Mean ± SD	185 (5mCi) ± 45
(range)	(110-300)
PSA (ng/ml) (n = 22; missing 7)	
Mean ± SD	43.04 ± 113.41
Median (range)	2.3 (0.003- 440)

CNR, contrast to noise ratio; SD, standard deviation; PSA, Prostate-Specific Antigen

**Figure 3:**
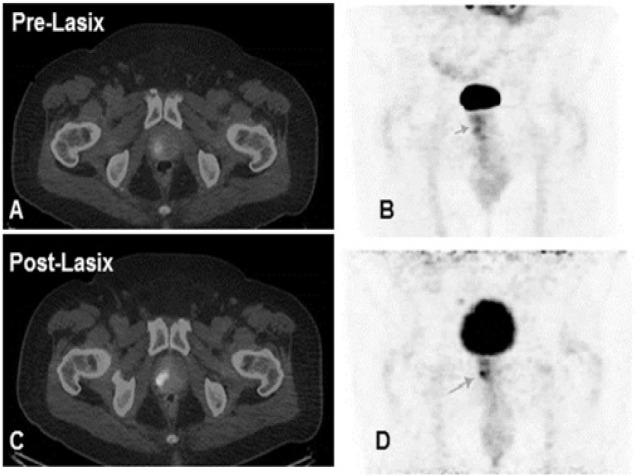
Fused PET/CT Images (A) and MIP (B) Showing Ill-Defined Lesion (Arrows) in the Prostate Gland. Post Lasix study in (C-D) showing better delineation of the lesion due to improvement of the image contrast

**Table 2:** Summary of Change in Reporting Opinion Post-Lasix (n=45)

Post-Lasix effect on opinion	No of cases (%)	Change type
Change in no of lesions	11 (24)	Major change
Change in confidence	5 (11)	Some change
Better delineation	10 (22)	Some change
Ureteric activity	7 (16)	Major change
Total cases with any change in opinion	33 (73)	(Major and Minor)
No change in decision	12 (27)	(No change)

**Figure 4:**
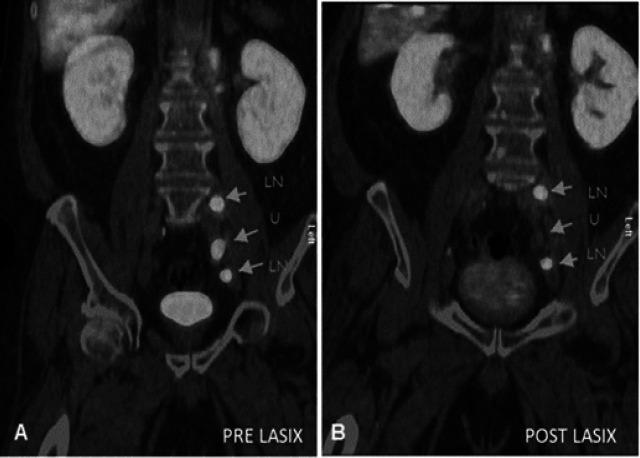
Coronal Fused PET/CT Images Showing Three Lesions in Athe Pre Lasix Study (Arrows), B) Post Lasix images show clearance of urine activity showing only two residual lesions. Urine uptake =U, Lymph node uptake=LN

**Figure 5:**
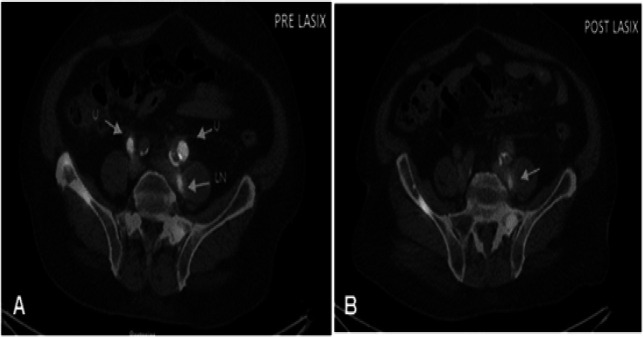
Transaxial Fused PET/CT Images (A) of the pelvis showing three focal lesions the in pre Lasix study (arrows), However, post Lasix images (B) showed clearance of activity from two areas labeled as ‘U’ leaving behind the true uptake in the lymph node (LN)

## Discussion

PCa has high incidence and recurrence rates in Kuwaiti population. The reason that the biochemical recurrence (after the initial curative treatment) always precedes clinical presentation which is 7–8 years on average, therefore there is a need of robust targeted imaging in PCa for prompt detection of subtle metastatic foci. The conventional diagnostic modalities like CT/MRI etc. has low/ poor diagnostic yield in asymptomatic patients. In the recent decade PSMA, has shown a measurable evidence in imaging of PCa and current guidelines recommend imaging with PSMA inhibitors using either 18F or ^68^Ga as imaging agent, with more acceptance of the later.

Although, there is a lot of evidence in favor of 18F-FDG PET-CT in restaging of PCa, however compared to ^68^Ga-PSMA PET-CT, the current guidelines recommend ^68^Ga-PSMA inhibitors in the imaging of PCa. Despite the fact that the scientists are working parallelly to evaluate possibly the better role of ^68^Ga-Fluciclivon PET-CT in imaging of PCa in recurrence setting, but still ^68^Ga-PSMA is the widely used tracer for imaging of PCa. (Wolfgang et al., 2017).

Current study focused on the evaluation of ^68^Ga-PSMA PET-CT imaging and its role in wide spectrum of possible equivocal lesions during the imaging of abdomen and pelvis, mainly around the kidneys, urinary bladder, prostate bed, retroperitoneal lymph nodes, urethral uptakes, peri-vesicle region and other possible artifacts. 

In current study, we objectively evaluated the significance of the diuretic assisted ^68^Ga-PSMA PET-CT in characterization of the metastatic/pathological lesions in imaging of PCa after 15±5 minutes. We used standard OSEM reconstruction in pre and post-Lasix images for visualization of the images which gives high quality images with low noise (P-value <0.05). 

A significant rise of CNR was observed in post-Lasix images i.e. 49.6 % ±24.5 (P-value 0.02) which in turn resulted in better delineation of the lesions in 10/45 (22%). A better confidence towards reporting of true positive lesions was observed in 5/45 (11%) of the cases, which is consistent with data reported by Fennessy et al., (2017) that post-furosemide imaging aid in the diagnostic interpretation of the images. Similarly, in 7/45 (16%) cases, ureteric activity mimicking metastatic lesions was washed-out during post-Lasix imaging (Figure 2). As the prostate is located distal to the bladder, lesions are influence by the scatter and spillover from the high activity in the bladder, resulting in the blurring of the lesions and lowering their contrast. Improved PSMA uptake overtime and clearance of ureteric activity by Lasix in these cases lead to better delineation of such foci. Wondergem et al., (2019) also showed that forced diuresis improves readability in terms of the improvement of lesion contrast and better delineation of the lesions. 

Post-Lasix imaging showed increase in the number of lesions in 11/45 (24%) post-Lasix studies when compared to pre-Lasix imaging (Figure 3) due to either better contrast or increase in SUV values. These results are in accordance to Maurer et al., (2016) who showed technical advantage of ^68^Ga-PSMA in lymph node staging when compare to the conventional imaging modalities like CT and MRI.

 In few cases, the ureter uptake was false positive which were cleared in the post-Lasix study (Figures 4, 5), however in other few cases, post-Lasix imaging did pick some true positive subtle lesions in these areas (Figure 3), due to better contrast, delayed incline in SUV values (due to increased PSMA uptake over time) and clearance of urine activity (Table 2). This post-Lasix incline in SUV values of up to 41% (range, 7.1%-100%) (P-Value < 0.05) is also one of the important confounding factor along with improvement in CNR in better delineation of the lesions, which augmented all physician’s opinion in differentiating true lesions from physiological urinary retention and on other occasions, few of the imperceptible lesions in the pre-Lasix study appeared as prominent metastatic foci in post-Lasix imaging.

Hence the statistical comparison of both methods with reference to change in number of lesions post diuretic study in all 90 images of (n=45) showed a significant statistical advantage of post-Lasix method over pre-Lasix method (P-value < 0.05) especially in characterization of the indeterminate/equivocal lesions in 16/45 (36%) of the cases and other parameters described in Table 2. This lead to change in their reporting quality and hence potentially significant impact in change of management of the patients, as per oncological perspective, each metastatic lesion counts. 

## Limitations of the study

The number of patients in current study were limited, however further studies on larger cohort are planned in near future. As current study was helped to reduced patient stay at hospital therefore due logistic reasons a delayed 2-3-hour scan without diuretics, for comparison, was not possible in current setting.

In conclusion, post diuretic ^68^Ga-PSMA PET-CT after 15±5 minutes has potential in improving the quality of reporting, by differentiating between ureteric/urethral activity from lymph node activity through increase in the CNR and SUV values, resulting in low false positive lesions. Although current Joint EANM and SNMMI guidelines also suggest a delayed imaging of 3-4 hour, but current results suggest that addition of a single Post-Lasix study at 15±5 minutes after the baseline scan, would eliminate the need for delay 3-4 hour imaging in a busy department, especially where a short lived tracer like Ga-^68^ and low image counts may lead to high image noise in delayed 3-4 hour images. The current study is a step forward in the harmonization of imaging protocols for PCa imaging and the data implies that addition of post-Lasix imaging has potential in improving the characterization of lesions, hence better reporting of a ^68^Ga-PSMA PET-CT with optimal management of the floor in terms of time management and reduced patient stay in the department. 
